# Reduction in Migratory Phenotype in a Metastasized Breast Cancer Cell Line via Downregulation of S100A4 and GRM3

**DOI:** 10.1038/s41598-017-03811-9

**Published:** 2017-06-14

**Authors:** Andy Chen, Luqi Wang, Bai-Yan Li, Jesse Sherman, Jong E. Ryu, Kazunori Hamamura, Yunlong Liu, Harikrishna Nakshatri, Hiroki Yokota

**Affiliations:** 1 Weldon School of Biomedical Engineering, Purdue University, West Lafayette, IN 47907 USA; 2 Department of Biomedical Engineering, Indiana University Purdue University Indianapolis, Indianapolis, IN 46202 USA; 3 Department of Pharmacology, School of Pharmacy, Harbin Medical University, Harbin, 150081 China; 4 Department of Mechanical Engineering, Indiana University Purdue University Indianapolis, Indianapolis, IN 46202 USA; 5 Department of Pharmacology, School of Dentistry, Aichi-Gakuin University, Nagoya, Japan; 6 Department of Medical and Molecular Genetics, Indiana University School of Medicine, Indianapolis, IN USA; 7 Department of Surgery, Simon Cancer Research Center, Indiana University School of Medicine, Indianapolis, IN 46202 USA

## Abstract

To investigate phenotypic and genotypic alterations before and after bone metastasis, we conducted genome-wide mRNA profiling and DNA exon sequencing of two cell lines (TMD and BMD) derived from a mouse xenograft model. TMD cells were harvested from the mammary fat pad after transfecting MDA-MB-231 breast cancer cells, while BMD cells were isolated from the metastasized bone. Compared to BMD cells, TMD cells exhibited higher cellular motility. In contrast, BMD cells formed a spheroid with a smoother and more circular surface when co-cultured with osteoblasts. In characterizing mRNA expression using principal component analysis, S100 calcium-binding protein A4 (S100A4) was aligned to a principal axis associated with metastasis. Partial silencing of S100A4 suppressed migratory capabilities of TMD cells, while Paclitaxel decreased the S100A4 level and reduced TMD’s cellular motility. DNA mutation analysis revealed that the glutamate metabotropic receptor 3 (GRM3) gene gained a premature stop codon in BMD cells, and silencing GRM3 in TMD cells altered their spheroid shape closer to that of BMD cells. Collectively, this study demonstrates that metastasized cells are less migratory due in part to the post-metastatic downregulation of S100A4 and GRM3. Targeting S100A4 and GRM3 may help prevent bone metastasis.

## Introduction

Tumor cells initiate their fate from non-tumor origins and continue to evolve via various transformations^1, 2^. While breast cancer cells originate as epithelial cells to form the primary tumor, they may acquire cellular motility and form a more invasive secondary tumor^3^. This metastatic alteration can be driven by epithelial-to-mesenchymal transition (EMT), in which the original epithelial nature is transformed into the migratory mesenchymal nature^4, 5^. However, many metastasized cells do not experience EMT, and the reverse transition, mesenchymal-to-epithelial transition, is speculated but not always confirmed^6^. Recent studies have indicated that metastasis may occur through the cooperative action of heterogeneous clusters of both epithelial and mesenchymal tumor cells^6, 7^. Since bone is the most frequent site of metastasis from breast cancer^8^, any phenotypic and genotypic differences before and after bone metastasis is critically important for determining the mechanism of metastasis as well as identifying diagnostic and therapeutic targets.

In this study, we focused on the differential migration and invasion abilities in two lines of breast cancer cells (TMD and BMD lines), which were harvested from a mouse xenograft model^9, 10^. In this model, MDA-MB-231 breast cancer cells were transfected into a mouse mammary fat pad, and TMD and BMD cells were recovered from the transfected site and metastasized bone, respectively. Using cDNA microarrays, genome-wide mRNA expression profiles were determined in these cells together with the parental MDA-MB-231 cells for predicting the genes involved in differential cellular motility. We also conducted DNA mutation analysis, focusing on exonic mutations that were potentially involved in the migratory behaviors of BMD and TMD cells. DNA from these cell lines were sequenced, and DNA variants in BMD cells were identified and characterized.

To extract metastasis-linked genotypic information from genome-wide mRNA expression profiles, principal component analysis (PCA) was applied. PCA is a mathematical procedure that decomposes mRNA expression levels into an orthogonal set of principal components (PCs)^11, 12^. Use of three cell lines in this study provided three PC axes, analogous to three degrees of freedom. Our primary interest herein is the differences in two cell lines (TMD vs. BMD cells). We focused on the first and second PC axes and located the set of genes that were likely to be involved in the differential migratory and invasive behaviors in the two cell lines.

Three *in vitro* assays were employed to characterize phenotypic differences in migratory and invasive behaviors, including a 2-dimensional motility assay^13^, a 3-dimensional invasion assay^14^, and a 3-dimensional spheroid assay^15^. Furthermore, a microfluidic assay was employed to characterize cellular motility in the presence and absence of Paclitaxel^16–18^.

## Results

### Higher migratory and invasive behavior of TMD cells than BMD cells

**Figure 1:**
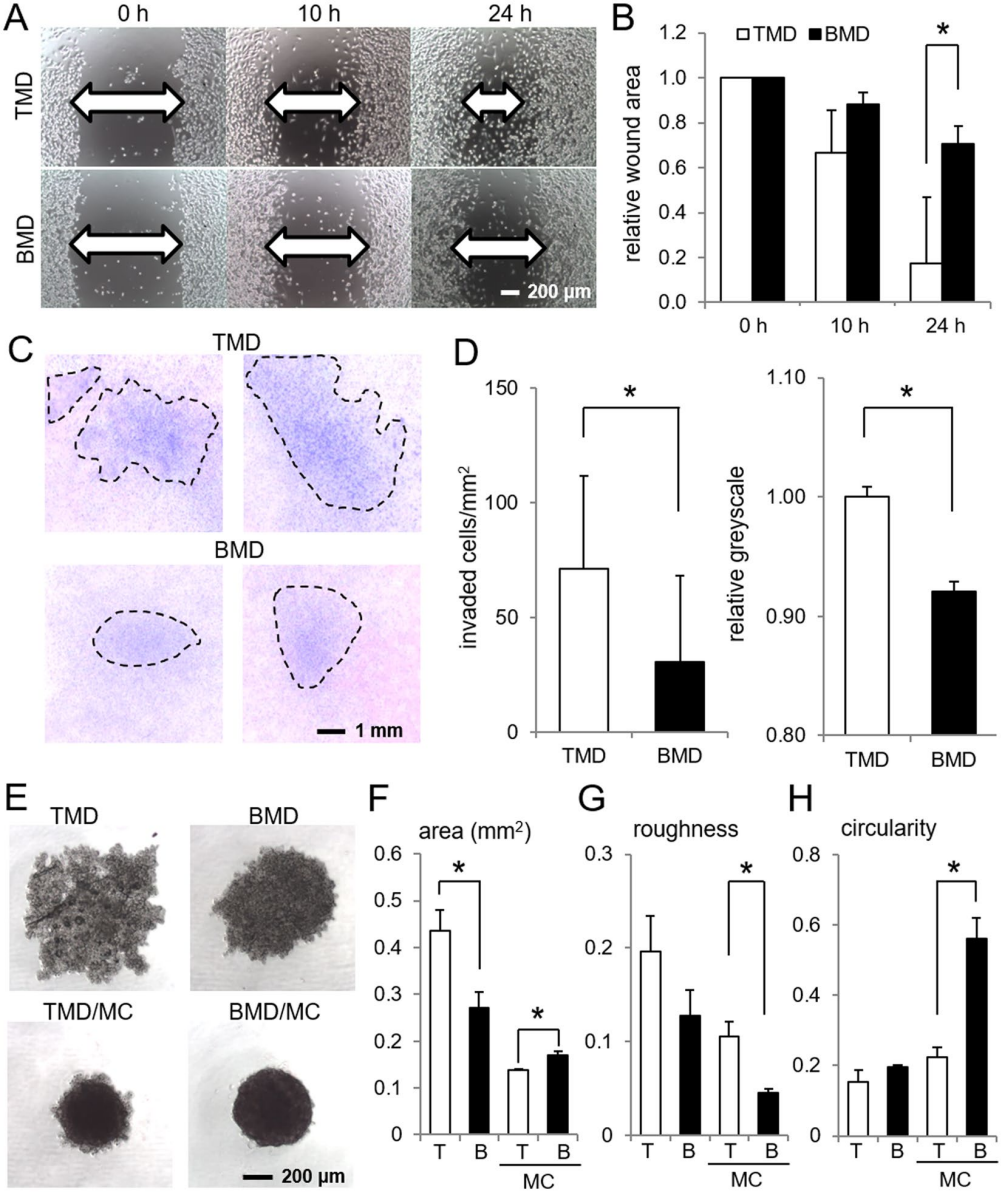
Phenotypic characterization of TMD cells and BMD cells. Of note, T = TMD cells, B = BMD cells, and MC = MC3T3 osteoblast-like cells. The single asterisk indicates *p* < 0.05. (**A**,**B**) Higher motility of TMD cells than BMD cells in a 2-dimensional scratch assay. (**C**,**D**) Higher invasion capability of TMD cells than BMD cells in a 3-dimensional invasion assay. (**E**) Spheroid formation of TMD and BMD cells with and without MC3T3 osteoblast-like cells. (**F**–**H**) Three spheroid parameters (area, roughness, and circularity, respectively) in TMD cells and BMD cells.

In a 2-dimensional cell motility assay, TMD cells exhibited a significantly higher motility than BMD cells (Fig. 1A,B). Furthermore, TMD cells showed a greater ability of invasion than BMD cells in a 3-dimensional invasion assay (Fig. 1C,D). In a 3-dimensional culture for spheroid formation, TMD cells formed a larger cluster of cell aggregates than BMD cells (Fig. 1E,F). When these cells were co-cultured with MC3T3 osteoblast-like cells, BMD cells formed a spheroid with a more circular and smoother surface than TMD cells (Fig. 1E–H).

### Differential expression of S100A4 highlighted in genome-wide principal component analysis

**Figure 2:**
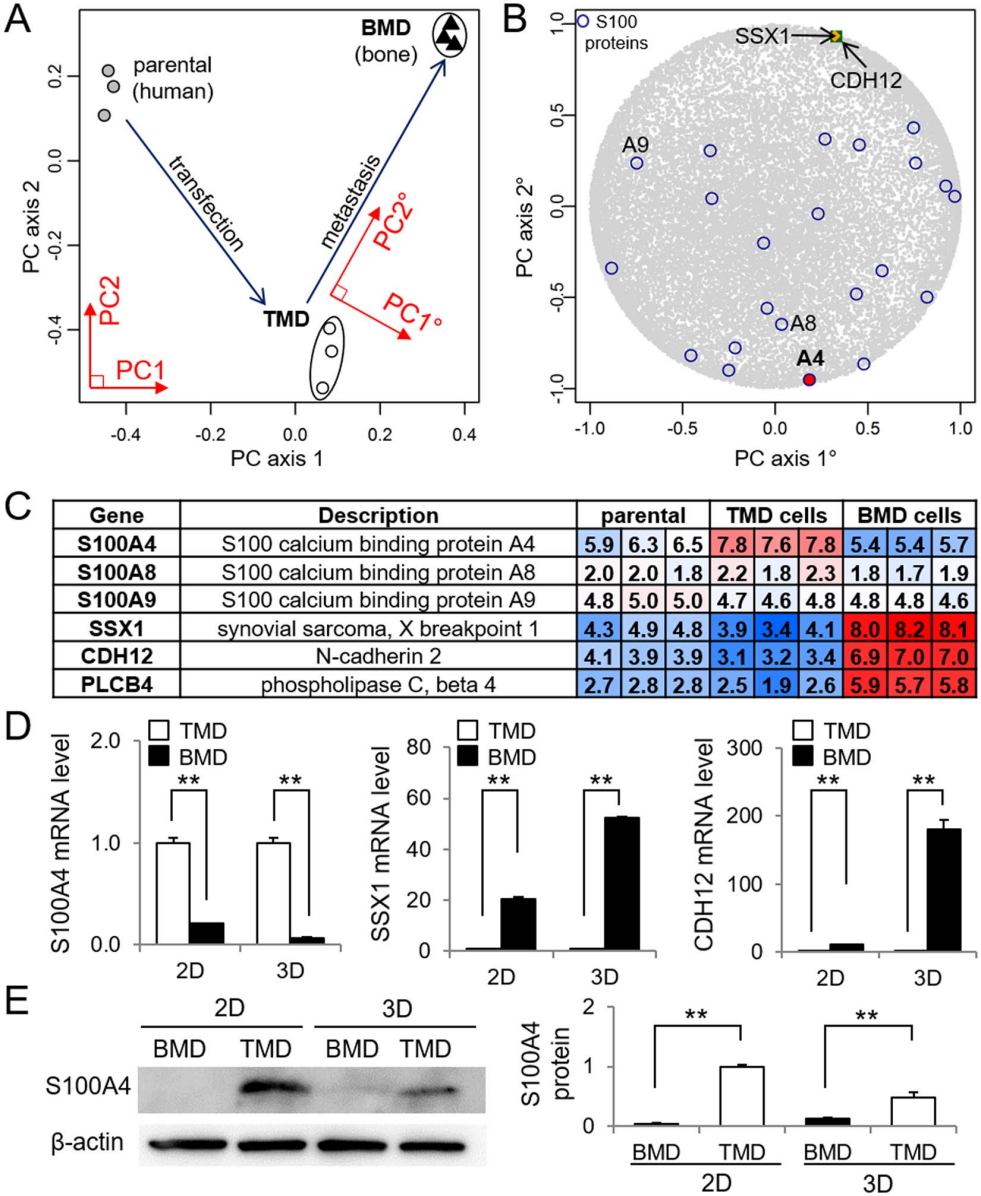
Genome-wide mRNA expression analysis of TMD cells and BMD cells. Of note, PC = principal component, 2D = 2-dimensional culture, and 3D = 3-dimensional spheroid culture. The double asterisks indicate *p* < 0.01. (**A**) Projection of three cell lines in the first/second PC array plane (PC1 and PC2). (**B**) Projection of S100 proteins and others in the PC gene plane (PC1° and PC2°). Note that the metastasis axis (PC2° axis) is ~20° tilted from the second PC axis (PC2 axis). (**C**) Representative genes whose mRNA expression levels are significantly different in TMD cells and BMD cells along the metastasis axis. (**D**) qPCR confirmation of mRNA expression levels of S100A4, SSX1, and CDH12 in TMD cells and BMD cells in 2-dimensional and 3-dimensional (spheroid) cultures. (**E**) Elevated protein level of S100A4 in TMD cells in 2- and 3-dimensional cultures. Full length blots are presented in Supplementary Figure 1.

Three cell lines (MDA-MB-231 parental cells, TMD cells and BMD cells) were located in the first and second PC plane, which was defined by performing singular value decomposition on a matrix of genome-wide mRNA expression (Fig. 2A). The first PC axis positioned TMD cells between the parental cells and BMD cells, while the second PC axis was ~20° rotated from the metastasis axis parallel to the line connecting TMD cells and BMD cells. We selected the genes whose expression level had a high contribution along the metastasis axis (Fig. 2B,C). Of note, the values in the color-coded panels indicate the microarray-derived relative expression levels on a logarithmic scale (base 2). Among such genes, qPCR confirmed that the expression level of three genes (S100A4, SSX1, and CDH12) are significantly different in TMD cells and BMD cells (Fig. 2D). Hereafter, we focused on S100A4 since its mRNA and protein levels were consistently higher in TMD cells than the parental and BMD cells in 2- and 3-dimensional cultures (Fig. 2D,E).

### Suppression of invasion behaviors of TMD cells by S100A4 siRNA

**Figure 3:**
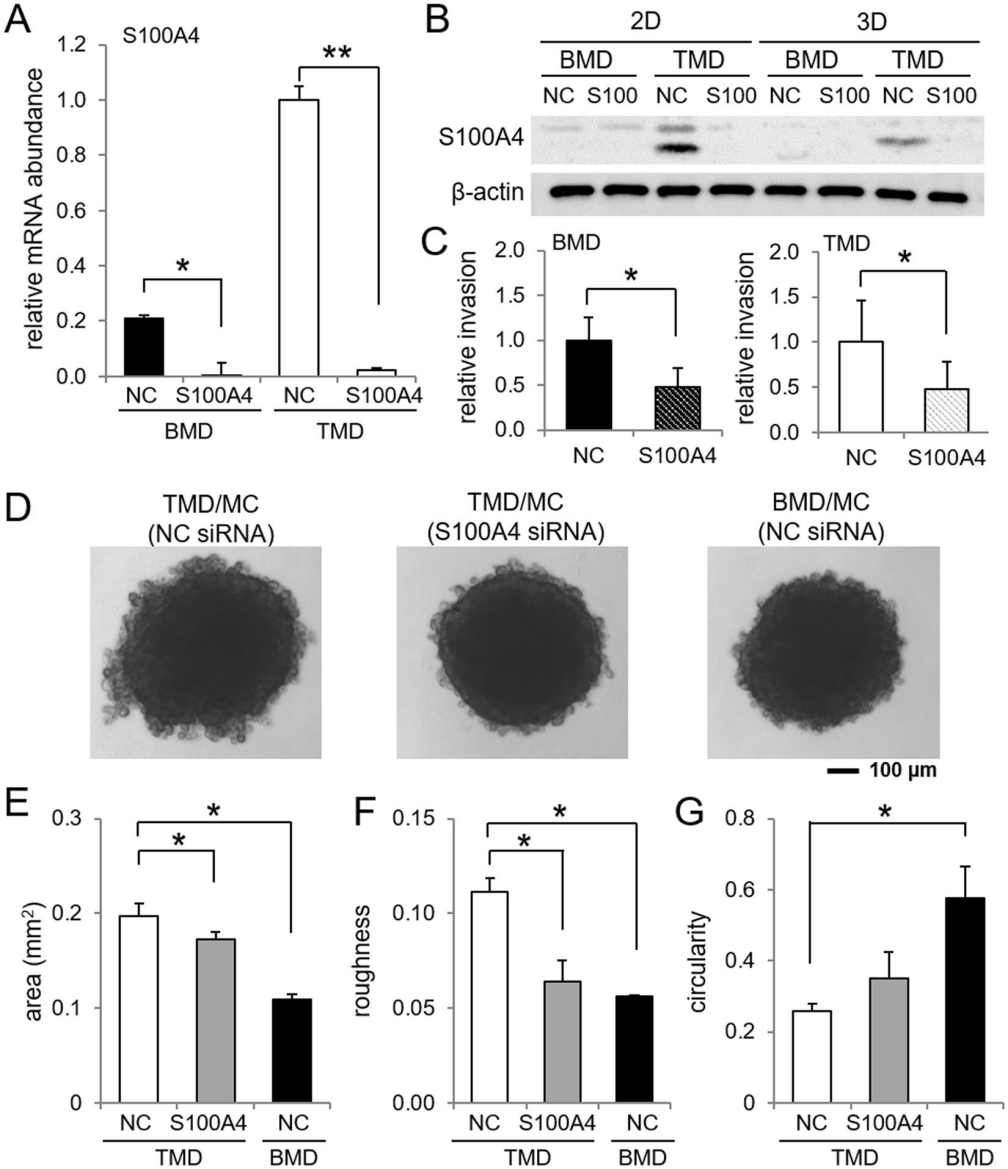
Effects of S100A4 siRNA in invasion capability and spheroid formation in TMD cells. Of note, NC = non-specific control siRNA, 2D = 2-dimensional culture, and 3D = 3-dimensional spheroid culture. The single and double asterisks indicate *p* < 0.05 and p < 0.01, respectively. (**A**,**B**) Reduction of S100A4 mRNA and protein levels by S100A4 siRNA, respectively. Full length blots are presented in Supplementary Figure 2. (**C**) Reduction in invasion capability by S100A4 siRNA in TMD cells and BMD cells. (**D**) Representative images of spheroids by TMD cells, S100A4 siRNA treated TMD cells, and BMD cells in the presence of MC3T3 osteoblast-like cells. (**E**–**G**) BMD-like transformation of three spheroid parameters (area, roughness, and circularity, respectively) by S100A4 siRNA.

A partial silencing of S100A4 by S100A4 siRNA reduced the mRNA and protein levels of S100A4 (Fig. 3A,B). This RNA interference reduced invasion capability in TMD cells and BMD cells in the 3-dimensional invasion assay (Fig. 3C). Furthermore, S100A4-treated TMD cells altered their phenotypic characteristics in spheroid formation (Fig. 3D). Compared to TMD cells treated with the non-specific siRNA, TMD cells treated with S100A4 siRNA significantly decreased their spheroid area as well as their surface roughness (Fig. 3E–G).

### Effects of GRM3 mutation in spheroid formation

**Figure 4:**
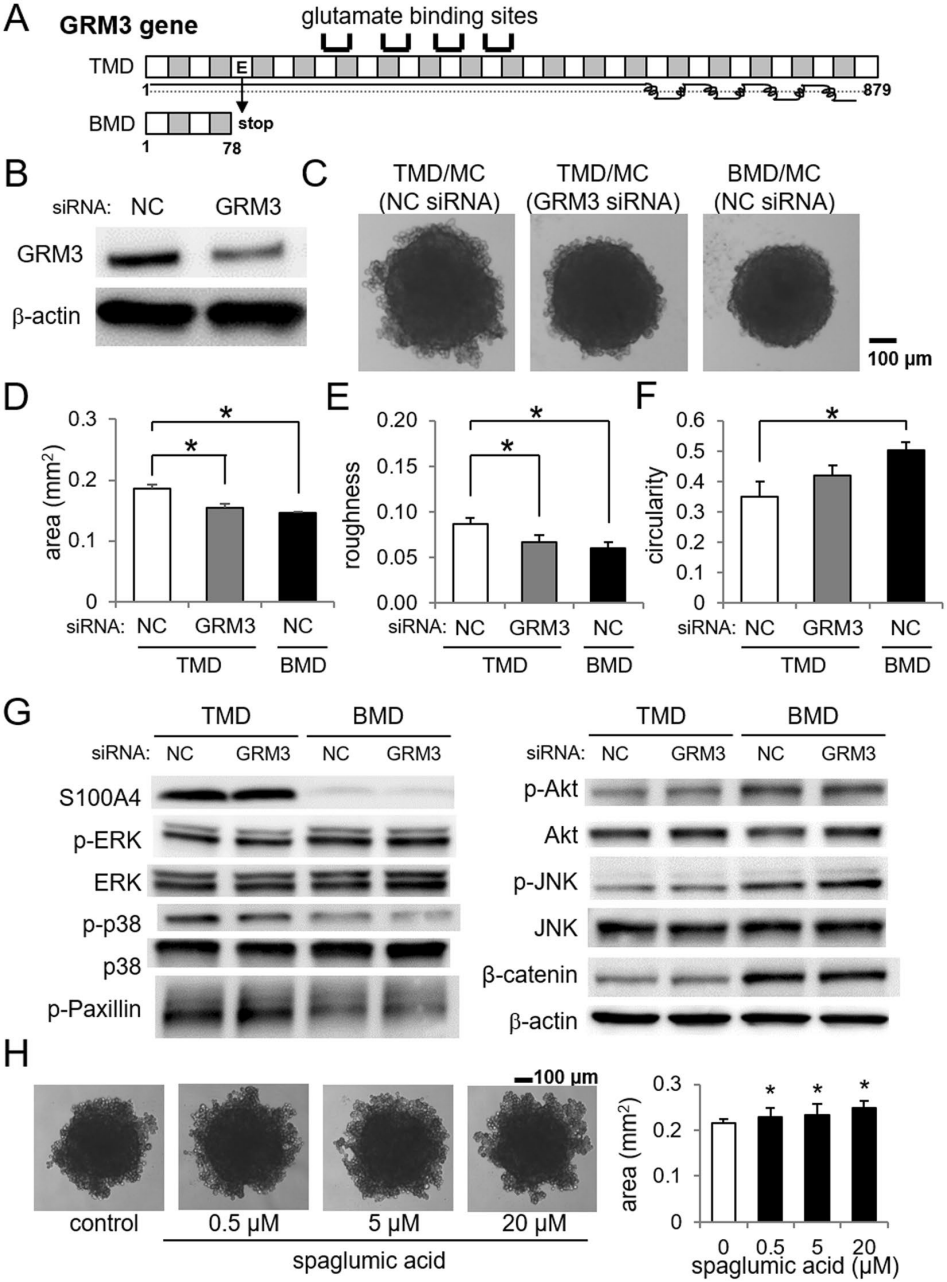
Effects of GRM3 in the spheroid shape. (**A**) Mutation of GRM3 in BMD cells. (**B**) Partial silencing of GRM3 using RNA interference. Full length blots are presented in Supplementary Figure 3. (**C**) Effects of GRM3 siRNA in the spheroid composed of TMD cells and MC3T3 cells. (**D**–**F**) Changes in the spheroid parameters by treatment with GRM3 siRNA in TMD/MC co-cultured cells. (**G**) Protein levels of S100A4, p-ERK, ERK, p-p38, p-38, p-Paxillin, p-Akt, Akt, p-JNK, JNK, β-catenin, and β-actin. Full length blots are presented in Supplementary Figure 4. (**H**) Effects of spaglumic acid treatment on the area of TMD and MC3T3 co-culture spheroids.

MuTect analysis of the exon sequences, comparing BMD variants to the TMD reference sequence, identified 4 stop-gains (GRM3, BHMT2, REXO1L1P, and FCGR1A) and 21 amino acid substitutions (0.42 to 0.70 allele frequency) that occurred in BMD cells but not TMD cells (Table 1). The mutation in GRM3 formed a truncated protein (78 amino acids instead of 879 amino acids in wildtype) in BMD cells (Fig. 4A). Partial silencing of GRM3 in TMD cells using RNA interference partially transformed the spheroid shape of TMD cells into that of BMD cells (Fig. 4B,C). More specifically, siRNA treatment significantly reduced the spheroid area and roughness of TMD cells (Fig. 4D–F). Western blot analysis revealed that compared to TMD cells, BMD cells had lower expression levels of S100A4, p-p38, and p-Paxillin, while their expression levels were higher for p-Akt, p-JNK, and β-catenin (Fig. 4G). In response to RNA interference with GRM3 siRNA, the level of p-p38 was lowered in BMD cells.

**Table 1:** Mutations in BMD cells.

symbol	description	allele freq.	base change	a.a. change
GRM3	glutamate receptor, metabotropic 3	1.00	G/T	E/stop
BHMT2	betaine-homocysteine S-methyltransferase 2	0.29	G/T	E/stop
REXO1L1P	REX1, RNA exonuclease 1 homolog-like 1	0.24	G/A	Q/stop
FCGR1A	Fc fragment of IgG Ia, receptor (CD64)	0.22	C/T	Q/stop
ANKRD20A4	ankyrin repeat domain 20 A4	0.70	A/G	S/G
ANKRD20A1	ankyrin repeat domain 20 A1	0.69	T/C	S/P
DMBT1	deleted in malignant brain tumors 1	0.69	A/G	H/R
ANKRD20A3	ankyrin repeat domain 20 A3	0.66	A/G	S/P
ANKRD20A3	ankyrin repeat domain 20 A3	0.62	C/A	S/I
ANKRD20A1	ankyrin repeat domain 20 A1	0.58	T/C	M/T
ANKRD20A1	ankyrin repeat domain 20 A1	0.55	A/G	S/G
ANKRD20A4	ankyrin repeat domain 20 A4	0.55	C/T	R/C
GGT2	gamma-glutamyltransferase 2	0.53	C/T	A/T
PLA2G2E	phospholipase A2, group IIE	0.51	G/A	R/C
LRRC37A2	leucine rich repeat containing 37, member A2	0.51	T/G	L/V
ZNF705D	zinc finger protein 705D	0.48	G/A	G/R
NUTM2D	NUT family member 2D	0.47	T/C	W/R
RASIP1	Ras interacting protein 1	0.47	C/G	A/P
SPATA31A6	SPATA31 subfamily A, member 6	0.46	C/T	A/T
KRTAP4–12	keratin associated protein 4–12	0.46	G/C	S/R
TAF4	TAF4 RNA polymerase II	0.46	C/A	A/S
CTAGE4	CTAGE 4	0.46	A/G	I/V
NPIPA5	nuclear pore complex interacting protein A5	0.45	A/G	V/A
USH2A	Usher syndrome 2 A	0.43	C/G	V/L
PLCB2	phospholipase C, beta 2	0.42	C/T	M/I

Co-culture spheroid formation assays were also performed with TMD cells in the presence of 0.5, 5, and 20 µM spaglumic acid, an agonist of GRM3. The spheroid area increased by treatment with spaglumic acid, indicating a more migratory environment (Fig. 4H).

### Involvement of integrin β, MMP9, and phosphodiesterases (PDEs)

**Figure 5:**
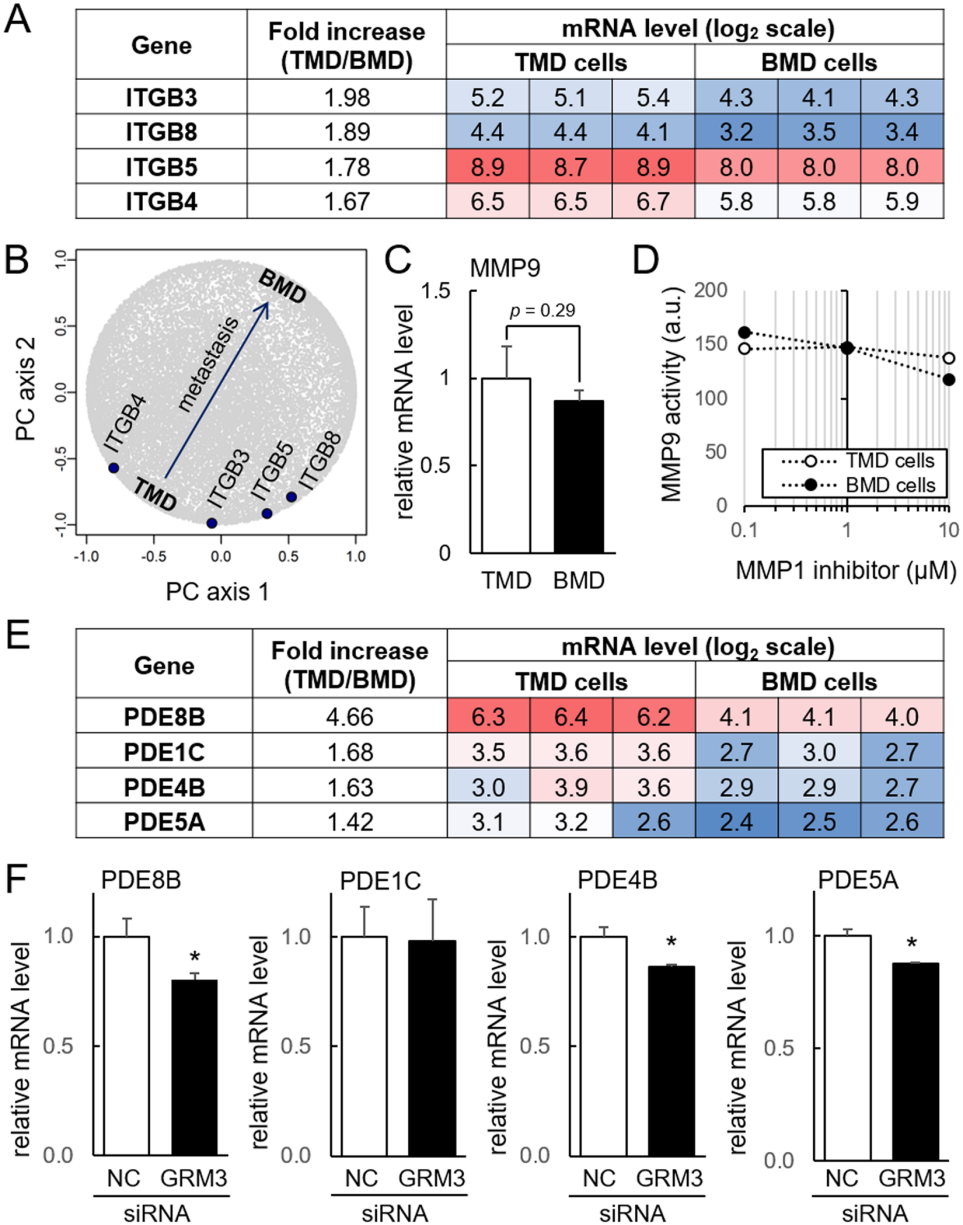
Elevated expression of integrin, MMP9, and phosphodiesterase (PDE) in TMD cells. (**A**,**B**) Elevated integrin β subunits (B3, B4, B5, and B8) in TMD cells. (**C**,**D**) Messenger RNA level and activity of MMP9. (**E**) Comparison of the mRNA levels of the selected PDEs (8B, 1C, 4B, and 5A) in TMD and BMD cells. (**F**) Reduction in the mRNA levels of the selected PDEs in TMD cells by GRM3 siRNA. NC = non-specific control siRNA.

We also examined any differential expression of integrin β and MMP9, which would potentially alter the microenvironment for cellular migration. Genome-wide expression analysis revealed that the mRNA levels of four members of integrin β family (B3, B4, B5, and B8) were significantly elevated in TMD cells (Fig. 5A,B). While the mRNA level of MMP9 was not of statistical significance, its activity level in cell culture medium was higher in TMD cells than BMD cells when 10 μM GM1489 (MMP1 inhibitor) was administered (Fig. 5C,D). Of note, the binding affinity of GM1489 is reported to be 0.2 nM for MMP1, and 100 nM for MMP9.

To evaluate a potential linkage of GRM3 to the inactivation of cAMP signaling followed by the stimulation of cellular migration, we determined the mRNA levels of PDEs in the microarray data. Compared to BMD cells, the selected PDEs were upregulated in TMD cells (Fig. 5E). In particular, the fold increase of PDE8B in TMD cells was 4.66, the highest increase among all PDEs. To further evaluate the role of GRM3 in cAMP signaling, we determined the mRNA levels of these PDEs (1C, 4B, 5A, and 8B) in the presence of non-specific control and GRM3 siRNA. The qPCR result showed that a partial silencing of GRM3 significantly downregulated the mRNA levels of PDE8B, PDE4B, and PDE5A in TMD cells (Fig. 5F).

### Effects of Paclitaxel in motility, S100A4 expression, and behavior in a microfluidic channel

**Figure 6:**
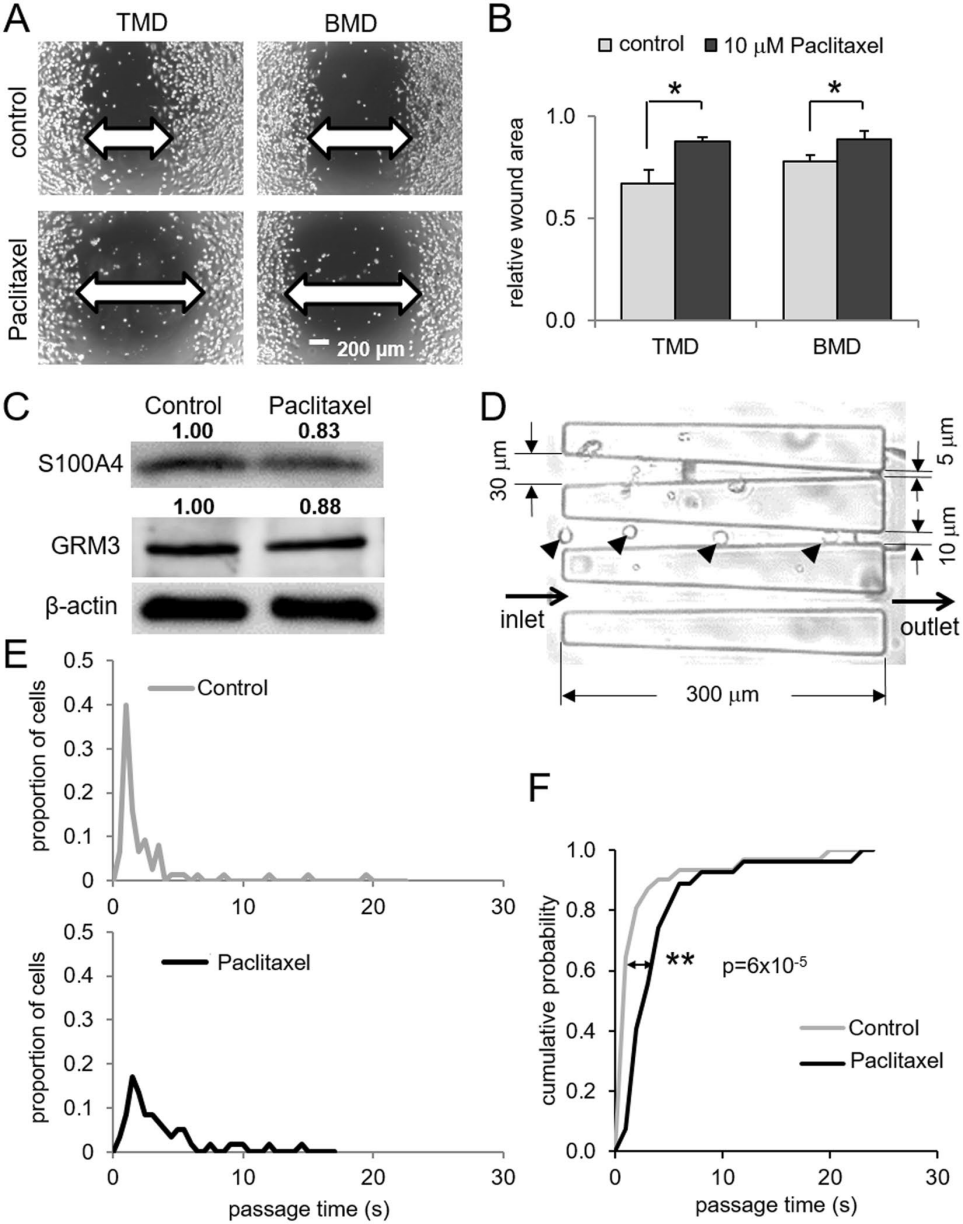
Effects of Paclitaxel in cellular motility, S100A4 expression, and passing behavior in a microfluidic channel. The single and double asterisks indicate *p* < 0.05 and *p* < 0.01, respectively. (**A**,**B**) Reduction in 2-dimensional cellular motility by 10 µM Paclitaxel in TMD cells and BMD cells. (**C**) Reduction in S100A4 protein level by 10 µM Paclitaxel in TMD cells. (**D**) Microfluidic channel for evaluating passing behaviors of TMD cells with and without Paclitaxel treatment. The arrow head indicates individual TMD cells in the channel. (**E**) Histogram of a passing time in a 300-µm channel for TMD cells (control) and Paclitaxel-treated TMD cells. (**F**) Longer passing time for Paclitaxel-treated TMD cells than control cells.

Paclitaxel is a popularly used drug for treatment of breast cancer. Both TMD cells and BMD cells were sensitive to 10 µM Paclitaxel and reduced their motility in a 2-dimensional scratch assay (Fig. 6A,B). Furthermore, the protein level of S100A4 was reduced by 10 µM Paclitaxel (Fig. 6C). Using a microfluidic channel that was connected to a hydrodynamic syringe pump, the passing behavior of TMD cells through a tapered channel was evaluated (Fig. 6D). The channels were 300-µm long with an inlet (30 µm) and several different sized outlets (5, 10, and 15 µm). We focused on an outlet with a 5-µm diameter and determined the passing time of cells through the channel for TMD cells (control) and Paclitaxel-treated TMD cells (Fig. 6E,F). The results showed that Paclitaxel-treated TMD cells required longer passing time on average than control cells.

## Discussion

PCA-based characterization of two cell lines revealed that the increased motility and invasion in TMD cells, tumor-derived cells, than BMD cells, harvested from bone metastasis, was linked to the elevated expression level of S100A4 calcium-binding protein and functionality of GRM3. A partial silencing of S100A4 suppressed the migratory behavior of TMD cells, and it further reduced cellular motility of BMD cells. Consistent with their migratory and invasive abilities, the more migratory TMD cells formed larger, looser, and less organized spheroids, while the metastatic BMD cells formed smaller, tighter, and more organized spheroids. Silencing S100A4 and GRM3 genes made the TMD cells form tighter spheroids, indicating potential roles in promoting metastasis in the bone microenvironment. A microfluidic assay with a tapered channel found that TMD cells passed more quickly, suggesting increased deformability. Paclitaxel treatment increased passing time in the channel, reduced S100A4, and inhibited TMD’s migratory ability. Our observation is consistent with an inhibitory role of Paclitaxel^18^ as well as a stimulatory role of S100A4 and GRM3 in cell adhesion and migration.

S100A4, a member of the S100 calcium binding protein family, was found to be a key gene in the migratory behavior of TMD cells that contributes to bone metastasis. The S100 protein family is composed of over 20 members in humans^19^, and many S100 proteins are known to be involved in tumor growth and metastasis^20^. In breast cancer, many S100 proteins are overexpressed. In particular, it is reported that S100A1 and S100A7 are linked to tumor growth, while S100A4 and S100A8 are linked to metastasis. S100A4 is active in the leading edge of migrating cancer cells^20^. Among the selected genes located at the extreme edges of the principal component plane, the mRNA level of S100A4 in TMD cells was significantly higher than not only BMD cells but also the parental cells. While the mRNA levels of SSX1 and CDH12 in TMD cells were smaller than in BMD cells, they were not significantly different from those of the parental cells. It is reported that S100A4 neutralizing antibody suppresses spontaneous tumor progression^21^. In this study, silencing S100A4 decreased the migratory character of TMD cells, suggesting that S100A4 is involved in promoting metastasis by the pre-metastatic TMD cells. Principal component analysis identified S100A4 as a metastasis-related gene, and further studies may explore its interactions with other genes on this bone metastasis axis.

GRM3, a glutamate receptor, has been considered a potential target for treating psychiatric disorders via modulating the response to glutamate as a neurotransmitter^22^. In this study, GRM3 was found to contribute to the migratory behavior of TMD cells. In our DNA sequencing analysis, GRM3 mutation in BMD cells changed the glutamate residue into a stop codon in the 78^th^ amino acids. The truncated GRM3 does not include glutamate binding sites or transmembrane domains, and it is apparently incapable of functioning as a G protein-coupled receptor. GRM3 is reported frequently mutated in melanoma, a common form of skin cancer, and mutated GRM3 increased cellular migration^23^. The upregulation of GRM3 is considered to lead to the inactivation of cAMP signaling followed by the stimulation of cellular migration^24^. Compared to BMD cells, the mRNA levels of several PDEs, including PDE8B, PDE4B, and PDE5A, were higher in TMD cells. The elevated expression of these PDEs in TMD cells is consistent with the reduction of cAMP signaling and promotion of cellular migration. These PDEs were downregulated in the presence of GRM3 siRNA, further supporting the notion that GRM3 is involved in the regulation of cAMP signaling via PDEs.

Among mitogen-activated protein kinases that are known to regulate cell migration^25^, the phosphorylation level of p38 (p-p38) was altered in two cell lines as well as by RNA silencing with GRM3 siRNA. Compared to TMD cells, less-migratory BMD cells showed a lower level of p-p38. Also, treatment of TMD cells with GRM3 siRNA lowered the level of p-p38. The result indicates that BMD’s reduction in cell migration is in part caused by the nonsense mutation of GRM3. Since treatment of TMD cells with GRM3 siRNA did not alter the level of S100A4, GRM3 does not directly regulate S100A4.

Cell migration and invasion also depend on interaction with the microenvironment. Four integrin β subunit genes were upregulated in TMD cells. Integrin β subunits are instrumental in facilitating feedback to cells about the surrounding mechanical environment. Their increased activity in tumor cells enhance their invasiveness^26^, and the elevated expression of ITGB3, ITGB4, ITGB5, and ITGB8 in TMD cells is consistent with their migratory capabilities. MMPs also alter the microenvironment by remodeling extracellular matrix, and the role of MMP2 and MMP9 in tumor migration is well documented^27^. While the activity of MMP9 was elevated in TMD cells, we also observed significant upregulation of MMP1 in BMD cells. Since BMD cells are harvested from the site of metastasis in bone, they may activate collagenases such as MMP1 for remodeling the novel microenvironment^28^.

A microfluidic experiment using a tapered channel demonstrated that TMD cells were less rigid and more deformable than BMD cells. Accumulating evidence indicates that cell rigidity differs depending on the developmental stage of tumor cells, and metastatic cells tend to be more deformable and softer than non-metastatic cells^29, 30^. It has been shown, for instance, that MDA-MB-231 cells are softer than MCF-7 non-tumor epithelial cells^31^. Using a microfluidic channel that was connected to a hydrodynamic syringe pump, the passing time of TMD cells in a narrowly tapered channel was characterized with and without Paclitaxel pretreatment. The longer passing time for Paclitaxel-treated cells indicates that Paclitaxel makes cells less deformable and/or more adhesive to the microfluidic channel. An understanding of the dynamical cellular process in this microfluidic assay may contribute to evaluating the role of chemotherapeutic agents for preventing metastasis through blood circulation. Furthermore, Paclitaxel’s inhibition of S100A4, potentially through modulating myosin assembly, may also help prevent migration and metastasis.

The phenotypic differences between tumor-derived (TMD) and bone metastasis-derived (BMD) cells may be explained by the cooperativity model of metastasis through a cluster of heterogeneous tumor cells. Accordingly, the cluster is composed of the mesenchymal-like TMD cells and the epithelial-like BMD cells. TMD cells are the drivers for migration away from the primary tumor site and invasion into the blood stream together with BMD cells. At the site of metastasis in a bone microenvironment, BMD cells have a higher chance of integration with surrounding bone cells because of their less migratory and stronger adherent phenotype. While S100A4 and GRM3 are two regulators that are associated with migration of tumor cells^20, 23^, decreased stiffness of TMD cells are consistent with their ability to invade and provide a path for epithelial-like BMD cells to the bone microenvironment.

There were several limitations to this study. We employed triple negative breast cancer cell lines instead of other types. Since bone metastasis is more frequent for ER-positive breast cancer cells, it is important to evaluate mRNA profiles and DNA variants in ER-positive cells^32^. TMD and BMD cells are likely to be composed of a variety of sub-populations with their unique DNA variants, and cell abilities of migration and invasion are dependent on other culture conditions besides 2- and 3-dimensional culture environments^33^. Finally, while BMD cells are reported to be linked to cytokine/hematopoietic stem cell signaling^9^, any role of BMD and TMD cells as cancer stem cells is yet to be elucidated. In summary, by analyzing differential mRNA expression profiles and exon variations in TMD/BMD cells, this study demonstrated that S100A4 and GRM3 induced TMD’s higher ability of cell motility and invasion, as well as a rougher, less circular surface in spheroids. Whether S100A4 and GRM3 can be utilized as therapeutic targets for bone metastasis from breast cancer is worth further pursuit.

## Materials and Methods

### Cell culture

MDA-MB-231 human breast cancer cells, TMD cells, and BMD cells, were grown in DMEM (Corning, Inc., Corning, NY, USA) and MC3T3 osteoblast-like cells were grown in αMEM (Gibco, Carlsbad, CA, USA). The culture media contained 10% fetal bovine serum (FBS) and antibiotics (50 units/ml penicillin, and 50 µg/ml streptomycin; Life Technologies, Grand Island, NY, USA). Cells were maintained at 37 °C and 5% CO_2_ in a humidified incubator. To measure the gene and protein expression levels in 2D, cells were seeded on 6-cm tissue culture dishes (Corning). To measure expression in three dimensions, 10^5^ cells were seeded in 100 µL culture media in U-bottom low-adhesion 96-well plates (S-Bio, Hudson, NH, USA), combining 6 wells per sample. After 48 h, cells were harvested.

### Motility assay and invasion assay

To evaluate 2-dimensional motility, a wound healing scratch motility assay was carried out. Cells were seeded in 6-well tissue culture plates, and on the next day, scratching was performed using a plastic pipette tip. The areas newly occupied with cells in the scratched zone were determined 10 h or 24 h after scratching using images obtained by a microscope quantified with Image J. To examine 3-dimensional invasion, Matrigel (100 µg/ml; BD Biosciences) was coated to the polyethylene terephthalate membrane (8-µm pore size, 23.1 mm in diameter, Falcon) and left to polymerize overnight. Cells (~5 × 10^5^/well) were seeded in the upper chamber. After 24 h, the cells on the membrane surface were stained with Giemsa (Sigma-Aldrich) and the number of cells was counted under the microscope.

### MMP9 activity assay

MMP9 activity in cell culture media was determined per manufacturer’s instructions using an activity assay kit with a fluorescent substrate cleaved by MMP-9 (SensoLyte 520 MMP9 Assay Kit, AnaSpec, Inc., Fremont, CA, USA). GM1489 (MMP1 inhibitor; EMD Millipore, Billerica, MA, USA) was supplemented to the reaction mixture at 0.1–10 μM to suppress nonspecific degradation of the substrate by MMP-1.

### Spheroid co-culture assay

To induce spheroid formation, TMD cells or BMD cells were cultured in a U-bottom low-adhesion 96-well plate in the presence and absence of MC3T3 cells (cell ratio 2:1, MC3T3:cancer cells; total cells 1 × 10^5^/well) in αMEM culture media^34^. After 48 h, microscope images were taken of the spheroid to be analyzed with ImageJ. A threshold was applied and the spheroid area was identified with the “Analyze Particle” function. Area and circularity of the identified object were measured directly with ImageJ. Roughness was calculated by fitting an ellipse to the spheroid and adding the areas of the spheroid outside the ellipse and the areas of the ellipse not within the spheroid.

### cDNA microarray and principal component analysis (PCA)

Using cDNA microarrays (Human Gene 2.0 ST, Affymetrix), genome-wide mRNA expression profiles were determined using RNA isolated with an RNeasy Plus Mini kit (Qiagen, Germantown, MD, USA) from 9 samples, including 3 samples each from 3 groups of cells (MDA-MB-231 parental cells, TMD cells, and BMD cells). In PCA, nine samples in three groups were positioned in the plane of the first and second principal component (PC) axes. In the plane defined by the first and second PC axes, we selected a specific axis (metastasis axis), which was parallel to the line connecting TMD cells to BMD cells. This metastasis axis was ~20° rotated from the second PC axis. Among S100 calcium binding proteins that are often involved in cancer metastasis, S100A4 gene was mapped at an extremum of the metastasis axis. Thus, its role for migratory behaviors of TMD/BMD cells was further evaluated.

### Real-time qPCR and Western blot analysis

Total RNA was extracted using an RNeasy Plus mini kit (Qiagen, Germantown, MD, USA) and reverse transcription was conducted with high capacity cDNA reverse transcription kits (Applied Biosystems, Carlsbad, CA, USA) to produce cDNA. Real-time qPCR was performed using Power SYBR green PCR master mix kits (Applied Biosystems) with PCR primers listed in Table 2. In Western blot analysis, cells were lysed in a radio-immunoprecipitation assay (RIPA) buffer. Isolated proteins were fractionated using 10% SDS gels and electro-transferred to polyvinylidene difluoride membranes (Millipore, Billerica, MA, USA). We used antibodies against S100A4, p-ERK, ERK, p-p38, p38, p-AKT, AKT, p-JNK, JNK, p-Paxillin, β-catenin (Cell Signaling), GRM3 (Abcam), and β-actin (Sigma). Protein levels were assayed using a SuperSignal west femto maximum sensitivity substrate (Thermo Scientific, Waltham, MA, USA).

**Table 2:** Real-time qPCR primers used in this study.

target	forward primer	backward primer
CDH12	5′- GAAATGTCTCCTGTGGGTGC -3′	5′-TTCCGGCTAATCCTCCAAGC -3′
GRM3	5′-CTTCACGGCTCCATTCAACC -3′	5′-GTTCCGGGACCAGTGGATAG -3′
PDE1C	5′-TTGAGTGTGGTGTGTGGTTCC -3′	5′-TGGCGACTCCATAGCTCAACAAG -3′
PDE4B	5′-GATGAGCCGATCAGGGAACC -3′	5′-CAGGTCTTCCAGCTCCTTGG -3′
PDE5A	5′-AGCAGTACCAGAGAGCCTCC -3′	5′-GCATTGACCATTTCTCTGGTGG -3′
PDE8B	5′-CGTGAAGCAGGTGTCTTCTG -3′	5′-ATAACCAGCTCTGTCGCAGG -3′
S100A4	5′-TTGGTGCTTCTGAGATGTGGG -3′	5′-CATGACAGCAGTCAGGATCAAC -3′
SSX1	5′-GAACTACAGGTGAGACTGCTCC -3′	5′-AAGGTGGGAGGGTGACTTTG -3′
GAPDH	5′-ATGGTGGTGAAGACGCCAGT -3′	5′-GCACCGTCAAGGCTGAGAAC -3′

### Knockdown of S100A4 and GRM3 by siRNA

Cells were treated with siRNA specific to S100A4 (5′- GCA UCG CCA UGA UGU GUA A -3′) (Life Technologies) or GRM3 (Dharmacon SMARTpool siRNA library). As a nonspecific control, a negative siRNA (Silencer Select #1, Life Technologies) was used. Cells were transiently transfected with siRNA in Opti-MEM I medium with Lipofectamine RNAiMAX (Life Technologies). Six hours later, the medium was replaced by regular culture medium. The efficiency of silencing was assessed with immunoblotting 48 h after transfection.

### DNA mutation analysis

Genomic DNA was extracted from BMD cells and TMD cells using QIAamp DNA mini kit (Qiagen). Exon capture analysis was conducted using the Complete Genomics platform (BGI, Cambridge, MA, USA). After receiving aligned sequences, high quality reads were filtered with samtools^35^, variants occurring in the BMD cells but not TMD cells were identified using MuTect (Broad Institute)^36^ and annotated using Variant Effect Predictor (Ensembl)^37^, followed by a manual assessment of their quality. We identified highly occurring variants, filtering by stop-gain and exonic missense mutations.

### TMD cells in a microfluidic channel

In a parallel array (20 mm in length and 1.23 mm in width), hydro-dynamically driven microfluidic channels, 300 µm in length, were designed and fabricated. Each channel has an inlet of 30 µm in width and an outlet of 5, 10, or 15 µm in width. TMD cells were pre-cultured in the presence and absence of 10 µM Paclitaxel for 24 h, and they were detached from the culture dish by trypsin prior to a flow experiment. In the microfluidic channel with 5 µm outlet, culture medium was introduced at a flow rate of 5 µl/min, and the passage time of individual cells from channel entry to exit was determined.

### Statistical analysis

Three or four independent experiments were conducted and data were expressed as mean ± S.D. Statistical significance was evaluated at *p* < 0.05, and the single and double asterisks in the figures indicate *p* < 0.05 and *p* < 0.01, respectively.

## Electronic supplementary material

Supplementary Information
