# Supplementary material for: Reduction in Migratory Phenotype in a Metastasized Breast Cancer Cell Line via Downregulation of S100A4 and GRM3
Source: Sci Rep. 2017 Jun 14;7:3459. doi: 10.1038/s41598-017-03811-9 (PMC5471271; doi:10.1038/s41598-017-03811-9)
Supplement: Supplementary file 1 — Supplementary Information [file 41598_2017_3811_MOESM1_ESM.pdf]

Supplementary Information

Reduction in Migratory Phenotype in a Metastasized Breast Cancer Cell Line via Downregulation of S100A4 and GRM3

Andy Chen[1], Luqi Wang[2, 3], Bai-Yan Li[3], Jesse Sherman[2], Jong E. Ryu[4], Kazunori Hamamura[5], Yunlong Liu[6], Harikrishna Nakshatri[7], and Hiroki Yokota[2]

[1]Weldon School of Biomedical Engineering, Purdue University, West Lafayette, IN 47907, USA
[2]Department of Biomedical Engineering, Indiana University Purdue University Indianapolis, Indianapolis, IN 46202, USA
[3]Department of Pharmacology, School of Pharmacy, Harbin Medical University, Harbin 150081, China
[4]Department of Mechanical Engineering, Indiana University Purdue University Indianapolis, Indianapolis, IN 46202, USA
[5]Department of Pharmacology, School of Dentistry, Aichi-Gakuin University, Nagoya, Japan
[6]Department of Medical and Molecular Genetics, Indiana University School of Medicine, Indianapolis, IN, USA
[7]Department of Surgery, Simon Cancer Research Center, Indiana University School of Medicine, Indianapolis, IN 46202, USA

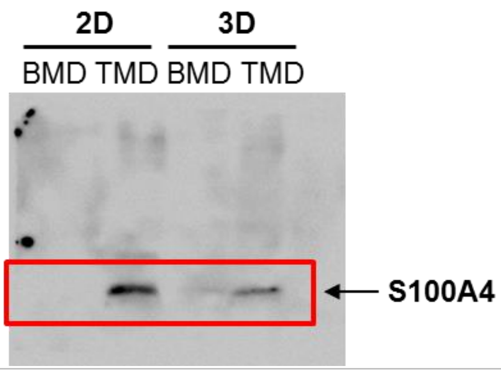

Supplementary Figure 1. Western blot gel image in Figure 2E.

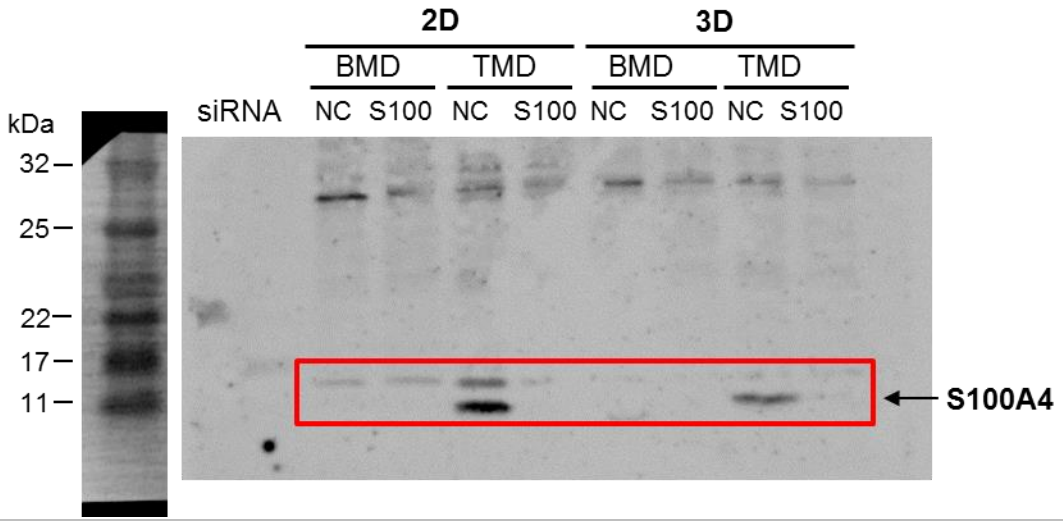

Supplementary Figure 2. Western blot gel image in Figure 3B.

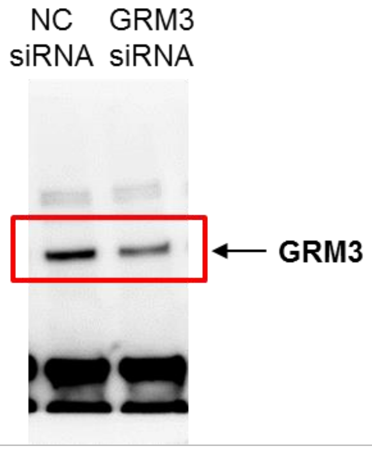

Supplementary Figure 3. Western blot gel image in Figure 4B.

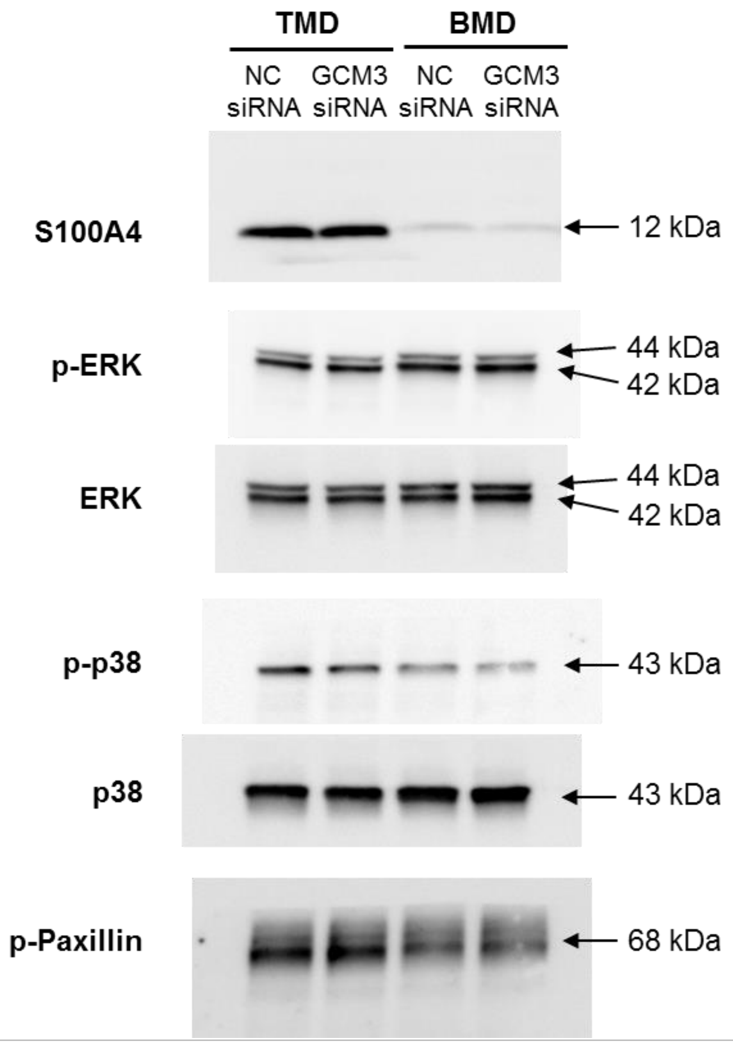

Supplementary Figure 4. Western blot gel image in Figure 4G.
